# Supplementary material for: Application of Near Infrared Spectroscopy for the Rapid Assessment of Nutritional Quality of Different Strawberry Cultivars
Source: Foods. 2023 Aug 29;12(17):3253. doi: 10.3390/foods12173253 (PMC10486686; doi:10.3390/foods12173253)
Supplement: Supplementary file 1 [file foods-12-03253-s001.zip › foods-2550567-supplementary.pdf]

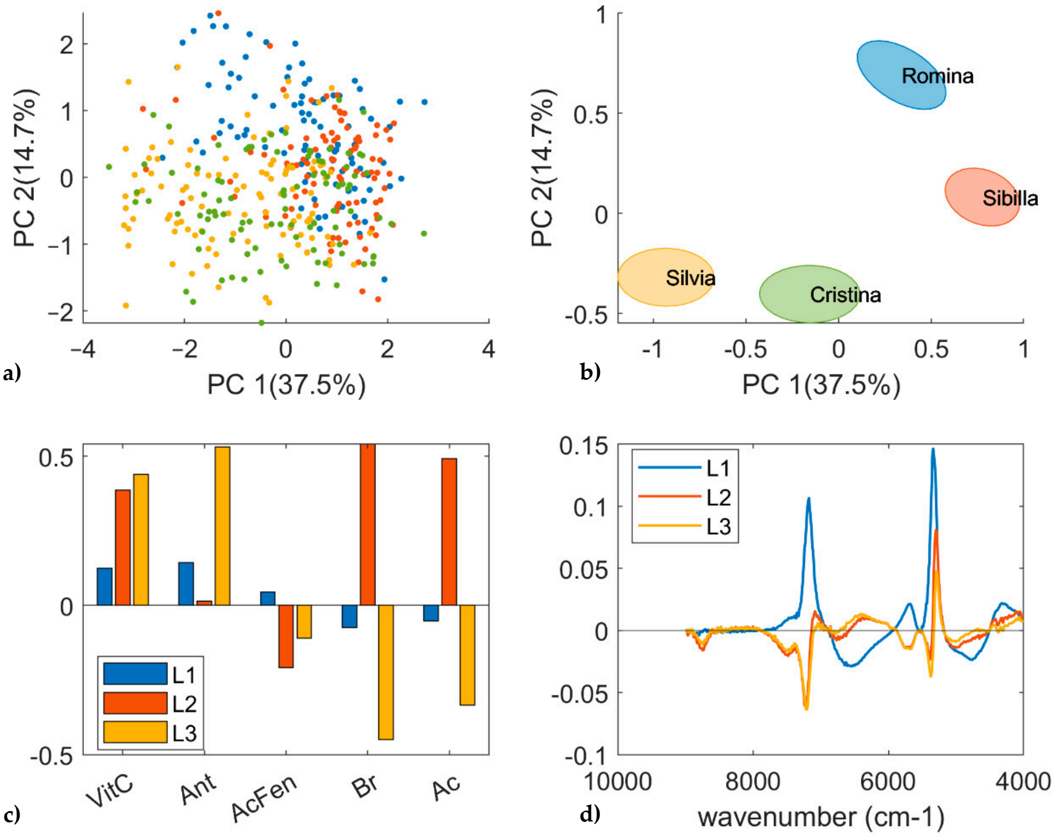

Figure S1: PCA score plot of PC1 vs PC2 computed on D-fused dataset with low-level approach (**a**) and with standard error ellipses for each cultivar (**b**). PCA loading plot of the three first PCs for D-Lab dataset (**c**) and D-NIR dataset (**d**).
